# Direct Actions of Kisspeptins on GnRH Neurons Permit Attainment of Fertility but are Insufficient to Fully Preserve Gonadotropic Axis Activity

**DOI:** 10.1038/srep19206

**Published:** 2016-01-12

**Authors:** Silvia León, Alexia Barroso, María J. Vázquez, David García-Galiano, María Manfredi-Lozano, Francisco Ruiz-Pino, Violeta Heras, Antonio Romero-Ruiz, Juan Roa, Günther Schutz, Milen Kirilov, Francisco Gaytan, Leonor Pinilla, Manuel Tena-Sempere

**Affiliations:** 1 Department of Cell Biology, Physiology and Immunology, University of Córdoba; CIBER Fisiopatología de la Obesidad y Nutrición, Instituto de Salud Carlos III; Instituto Maimónides de Investigación Biomédica de Córdoba/Hospital Universitario Reina Sofia (IMIBIC/HURS), 14004 Córdoba, Spain; 2 Molecular Biology of the Cell I, German Cancer Research Center, Heidelberg, Germany

## Abstract

Kisspeptins, ligands of the receptor, Gpr54, are potent stimulators of puberty and fertility. Yet, whether direct kisspeptin actions on GnRH neurons are sufficient for the whole repertoire of their reproductive effects remains debatable. To dissect out direct vs. indirect effects of kisspeptins on GnRH neurons *in vivo*, we report herein the detailed reproductive/gonadotropic characterization of a Gpr54 null mouse line with selective re-introduction of Gpr54 expression only in GnRH cells (Gpr54^−/−^Tg; rescued). Despite preserved fertility, adult rescued mice displayed abnormalities in gonadal microstructure, with signs of precocious ageing in females and elevated LH levels with normal-to-low testosterone secretion in males. Gpr54^−/−^Tg rescued mice showed also altered gonadotropin responses to negative feedback withdrawal, while luteinizing hormone responses to various gonadotropic regulators were variably affected, with partially blunted relative (but not absolute) responses to kisspeptin-10, NMDA and the agonist of tachykinin receptors, NK2R. Our data confirm that direct effects of kisspeptins on GnRH cells are sufficient to attain fertility. Yet, such direct actions appear to be insufficient to completely preserve proper functionality of gonadotropic axis, suggesting a role of kisspeptin signaling outside GnRH cells.

Kisspeptins, the products of the *Kiss1* gene that bind the receptor Gpr54 (aka, Kiss1R), have been unanimously recognized as indispensable elements in the neuroendocrine control of puberty and reproduction^1^,^2^. This essential function, which is now documented by a wealth of experimental data from different species, was initially disclosed by phenotypic observations in patients and mice harboring inactivating mutations of Gpr54, which displayed lack of puberty and hypogonadotropic hypogonadism (HH); features that were later confirmed in Kiss1-deficient individuals. Compelling evidence gathered during the last decade points out that kisspeptins primarily operate at central (hypothalamic) levels to stimulate GnRH secretion, and thereby reproductive function^1^,^2^.

The capacity of kisspeptins to act directly on GnRH neurons is supported by the observations that GnRH neurons express Gpr54, and that kisspeptins are able to activate (c-fos expression) and to induce potent depolarization responses in GnRH neurons^3^, even after blockade of synaptic transmission^4^. In addition, Kiss1 neurons have been shown to send projections to GnRH neurons^5^. In good agreement, kisspeptins have been reported to stimulate GnRH secretion *ex vivo* and *in vivo* in various species, including rodents, sheep and primates, while the potent LH-releasing effects of kisspeptins were blocked by pre-treatment with GnRH antagonists^1^. The latter observations, however, do not preclude additional sites of action of kisspeptins, on afferents to GnRH neurons rather than GnRH neurons themselves. In fact, the number of synaptic contacts between Kiss1 and GnRH neurons appears to be surprisingly low and only a limited fraction of Kiss1 neurons from the arcuate nucleus (<20%) projects to the preoptic area, where most of GnRH cell bodies are located^5^. While these features do not exclude the possibility of contacts at the level of GnRH nerve terminals in the median eminence, or non-synaptic communication (e.g., via volume transmission), they are also compatible with indirect effects of kisspeptins in the central control of GnRH neurosecretion.

Indeed, although electrophysiological analyses have unambiguously documented that kisspeptins can directly activate GnRH neurons, they also evidenced that kisspeptins are capable to activate non-GnRH neurons in the medial preoptic area and that part of GnRH neuronal responses to kisspeptins is blunted following blockade of fast synaptic transmission, mediated mostly by ionotropic γ-aminobutyric acid (GABA) and glutamate receptors^6^. The physiological relevance of such indirect, afferent-mediated effect of kisspeptins on GnRH neurons is reinforced by the fact that it seems dependent on proper estrogen input^6^, which is mandatory for manifestation of full LH responses to kisspeptins *in vivo*^7^. In fact, kisspeptins have been shown to modulate GABA and glutamate transmission to GnRH neurons, in an estrogen-dependent manner^8^. In the same vein, LH responses to kisspeptin *in vivo* were blunted following inhibition or activation ionotropic glutamate or GABA receptors, respectively^9^. Moreover, kisspeptin-Gpr54 signaling has been identified as key element in the control of preoptic neurons synthesizing the gaseous transmitter, nitric oxide (NO), which participates in the regulation of GnRH secretion^10^. In addition, kisspeptin pathways may also mediate at least part of the effects of other central regulators of GnRH neurons, as documented by the fact that LH responses to NMDA were largely suppressed in Gpr54 null mice^11^, which were also totally unresponsive to different agonists of tachykinin (TK) receptors, NK1R, NK2R and NK3R, mediating the effects of substance P, neurokinin A (NKA) and NKB, respectively^12^. Altogether, these data illustrate an intricate, bidirectional interplay between kisspeptins and other central transmitters that goes beyond a mere direct action of kisspeptins on GnRH neurons.

In addition to central effects, expression of Kiss1/kisspeptins and Gpr54 has been reported at other levels of the hypothalamic-pituitary-gonadal (HPG) axis, although the physiological relevance of kisspeptin signaling outside the brain remains debatable^1^,^13^. Admittedly, the central effects of kisspeptins clearly dominate their reproductive actions, but it is also tenable that, due their potent nature, these might obscure the eventual peripheral effects of kisspeptins, which may play a role in the physiological tuning of gonadal function. Indeed, expression of the elements of the Kiss1 system has been reported in the gonads, and very recent evidence suggests that local kisspeptin signaling might play a role in testicular testosterone secretion and Leydig cell function^14^, as well as in oocyte survival and, eventually, ovarian ageing^15^,^16^. In addition, kisspeptins are expressed in the rat oviduct and mouse uterus^17^,^18^, and uterine kisspeptin signaling might directly regulate embryo implantation^18^.

In an attempt to elucidate the relative importance of direct (vs. indirect or independent) effects of kisspeptins on GnRH neurons, a mouse model was recently generated where, upon a Gpr54 deficient background, expression of kisspeptin receptors was selectively rescued in GnRH-expressing cells, namely, the Gpr54^−/−^Tg rescued mouse^19^. By using this model of GnRH-specific Gpr54 (re)expression, it was recently proposed that kisspeptin actions on GnRH neurons are sufficient to attain fertility. Yet, important aspects of the functionality of the HPG axis were not addressed in those initial studies, which fell short in providing conclusive evidence for the lack of more subtle perturbations of the reproductive system, of considerable pathophysiological potential. Our detailed neuroendocrine phenotyping of the Gpr54^−/−^Tg rescued mouse evidences that, although sufficient for puberty to proceed and to achieve reproductive capacity, direct kisspeptin effects on GnRH neurons seem to be insufficient to fully preserve the proper functionality of gonadotropic axis, which is compatible with a reproductive role of kisspeptin signaling outside GnRH cells.

## Results

### Reproductive phenotypic characterization of the Gpr54^−/−^Tg rescued mouse

In order to address the physiological relevance of direct vs. indirect effects of kisspeptins on GnRH neurons, we conducted a series of phenotypic and neurohormonal analyses in both males and females of a recently generated GnRH cell-specific Gpr54 expressing (rescued) mouse line showing selective (re)expression of Gpr54 in GnRH cells, upon a Gpr54 null background^19^. In this mouse line, effective re-expression of Gpr54 was solidly documented by GFP detection (used as marker for transgene expression) in GnRH neurons, and the rescue of GnRH firing responses to kisspeptin-10 (totally absent in global Gpr54^−/−^ mice), even after pre-treatment with an chemical cocktail to block any presynaptic inputs^19^. For comparative purposes, in our studies, three genotypes were evaluated: WT, Gpr54^−/−^Tg and Gpr54^−/−^ (null) mice. In keeping with a previous report, Gpr54^−/−^Tg mice of both sexes attained reproductive maturation, as evidenced by complete vaginal opening and balano-preputial separation, and were fertile as young adults^19^; in contrast, Gpr54^−/−^ null mice did not go through puberty and were infertile.

**Figure 1:**
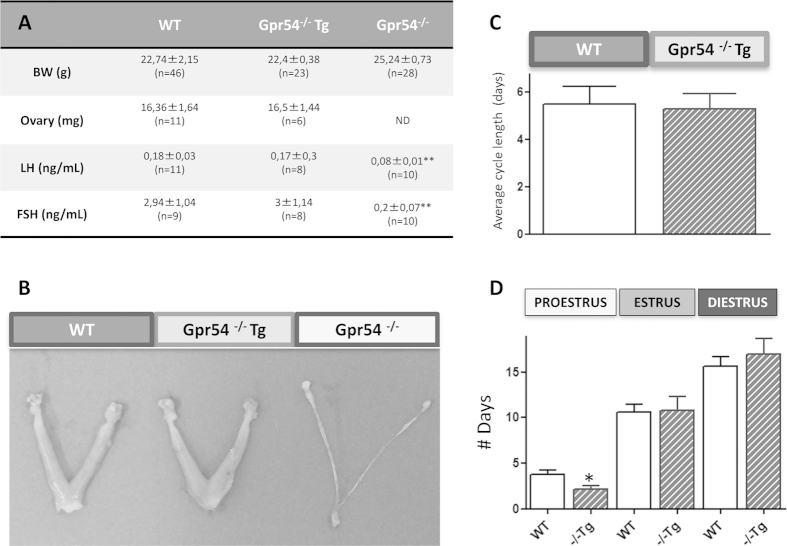
Female reproductive phenotype of the Gpr54^−/−^Tg rescued mouse. Body and gonadal weights, as well as serum LH and FSH levels in adult WT, Gpr54^−/−^Tg rescued and Gpr54^−/−^ null female mice are shown in panel (**A**). In addition, in panel (**B**), representative photographs of the ovaries of uteri of the three genotypes are presented. Finally, studies of the dynamics of estrous cyclicity are presented in panels (**C**,**D**); these included the assessment of the number of days per each stage of the estrous cycle over a period of 30-days, and the average cycle length during this period in WT and rescued female mice. Note that Gpr54^−/−^ null females did not display vaginal opening and, hence, no cytological analyses were conducted in this genotype. *P < 0.05 and **P < 0.01 vs. corresponding WT groups (Student t-tests or ANOVA followed by Student-Newman-Keuls multiple range test).

**Figure 2:**
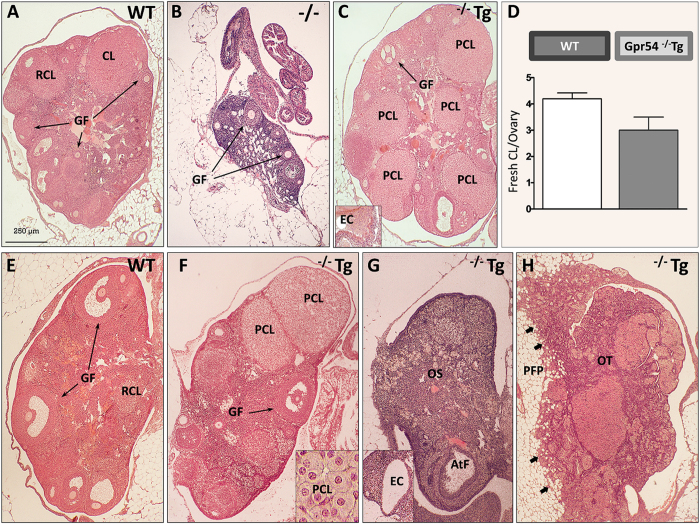
Alterations in ovarian ultrastructure in the Gpr54^−/−^Tg rescued female mouse. A series of representative ovarian sections from WT, Gpr54^−/−^Tg rescued and Gpr54^−/−^ null female mice, stained with hematoxylin and eosin, are shown. At 4 months of age, WT mice showed normal cycling ovaries with growing follicles (*GF*), and fresh (*CL*) and regressing (*RCL*) corpora lutea (panel **A**). In contrast, Gpr54^−/−^ null mice showed small ovaries, with pre-antral follicles as the most advanced stage and absence of corpora lutea (panel **B**). In turn (see panel **C**), Gpr54^−/−^Tg mice showed cycling ovaries with normal features, except for the presence of occasional persistent corpora lutea (*PCL*) and epithelial cysts (*EC* in the *inset*); yet, the number of fresh CL per ovary was not significantly different from WT (see panel **D**). At 12 months of age, WT mice showed normal cycling ovaries (see panel **E**), whereas age-matched Gpr54^−/−^Tg rescued mice (see panels **F**–**H**) had ovaries with pathological changes reminiscent of ovarian ageing, such as persistent corpora lutea (*PCL*, see **F**) and ovarian atrophy (see **G**), which presented as dense ovarian stroma (*OS*) and occasional atretic follicles (*AtF*), as well as epithelial cysts (*EC* in the *inset*). In addition, one Gpr54^−/−^Tg rescued female showed an ovarian tumor (see **H**), involving the whole ovary (*OT*) and invading (*arrows*) the peri-ovarian fat pad (*PFP*).

In young adult (3–4 month-old) Gpr54^−/−^Tg female mice, body weight (BW) gain, as well as ovarian and uterus weights were similar to those of WT animals (Fig. 1A). In contrast, female Gpr54^−/−^ null mice of the same age showed a significant increase in adult BW and dramatically reduced ovarian and uterus weights; in fact, reproductive organs were macroscopically very small in female Gpr54^−/−^ mice (Fig. 1B), and fresh accurate dissection for weighing was not possible. Similarly, basal LH and FSH levels were similar between WT and Gpr54^−/−^Tg rescued female mice, while Gpr54^−/−^ null females displayed a striking suppression of circulating LH and FSH concentrations (Fig. 1A). In the same vein, young adult (4-mo-old) Gpr54^−/−^Tg females showed preserved estrous cyclicity, except for a slight decrease in % of proestrus (Fig. 1C,D), and overtly normal ovarian histology, with abundant growing follicles and the presence of corpora lutea; these morphological features were similar to those of age-matched WT females, which showed normal cyclic ovaries with follicles at different stages of development and corpora lutea (Fig. 2A,C). In clear contrast, Gpr54^−/−^ null mice of the same age displayed small ovaries, lacking corpora lutea and with preantral follicles as the most advanced stage (Fig. 2B).

Yet, in some young adult Gpr54^−/−^Tg females, persistent regressing corpora lutea and occasional epithelial cysts were observed (Fig. 2C). In addition, a non-significant decline in the number of fresh corpora lutea per ovary was detected in rescued female mice (Fig. 2D). Of note, differences in the ovarian phenotype became more evident with incipient ageing, as evidenced by morphometric analyses in a subset of WT and Gpr54^−/−^Tg mice (N = 5/each genotype). Thus, 12-mo-old WT female mice showed abundant growing follicles, as well as fresh and regressing corpora lutea (Fig. 2E), indicative of normal cyclicity. In contrast, only 40% of Gpr54^−/−^Tg mice had corpora lutea of the current cycle, while a majority showed signs of persistent anovulation (namely, lack of fresh corpora lutea). Furthermore, this genotype showed discernible morphological alterations in the ovary, such as large persisting corpora lutea (Fig. 2F), epithelial cysts (Fig. 2G), atrophy (Fig. 2G), and even a tumor involving the whole ovary and invading peri-ovarian fat pads (Fig. 2H). While detailed quantitative analyses were not conducted at this age, these morphological alterations are clearly reminiscent of the histopathological ovarian changes detected during reproductive senescence in female rodents.

**Figure 3:**
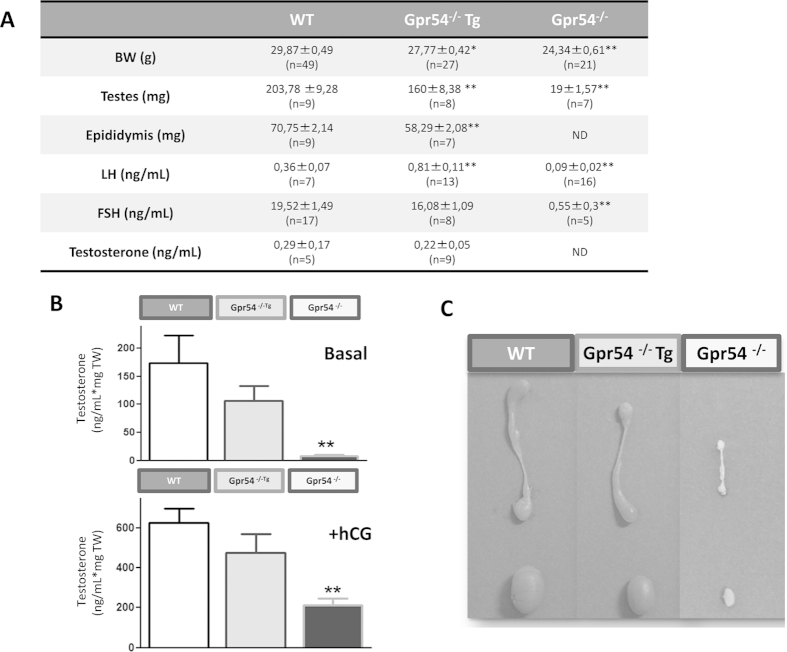
Male reproductive phenotype of the Gpr54^−/−^Tg rescued mouse. Body, testis and epididymis weights, as well as serum LH, FSH and testosterone levels in adult WT, Gpr54^−/−^Tg rescued and Gpr54^−/−^ null male mice are shown in panel (**A**). In addition, testosterone secretion, at basal conditions and after stimulation with effective concentrations of hCG, by static incubations testicular explants *in vitro* is presented in panel (**B**). Finally, in panel (**C**), representative photographs of the testis and epididymis of the three genotypes are presented. *P < 0.05 and **P < 0.01 vs. corresponding WT groups (Student t-tests or ANOVA followed by Student-Newman-Keuls multiple range test).

In young adult (3–4 month-old) males, Gpr54^−/−^Tg mice displayed an intermediate phenotype on different somatic and reproductive indices, as compared with aged-matched WT and Gpr54 null mice (Fig. 3). Thus, Gpr54^−/−^ males showed lower BW (~80% vs. WT) and dramatically decreased testicular and epididymis weights (Fig. 3A,C). Alike, Gpr54^−/−^Tg rescued males had smaller testes and epididymis, and lower BW vs. WT mice. Yet, body and testicular/epididymis weights were significantly higher in rescued males than in KO animals; in fact, testes and epididymis were reduced by ~20% in Gpr54^−/−^Tg males, whereas in Gpr54 null mice the drop in testis weight was >90% (Fig. 3A,C). Similarly, moderate but detectable alterations in reproductive hormone levels were observed in Gpr54^−/−^Tg males; yet, these were less dramatic than those seen in Gpr54 null mice. Thus, while young adult Gpr54^−/−^Tg male mice had similar basal FSH levels, the circulating LH levels were significantly increased in this genotype (Fig. 3A). Basal T levels *in vivo*, and especially total basal and hCG-stimulated T secretion by testicular explants *ex vivo* showed a trend for (non-significant, ~25%) decrease in Gpr54^−/−^Tg males (Fig. 3B). However, Gpr54 null mice showed more dramatic changes in T, LH and FSH serum level, which collapsed to nearly undetectable values for T and FSH secretion, as well as T release *ex vivo* (Fig. 3A,B).

**Figure 4:**
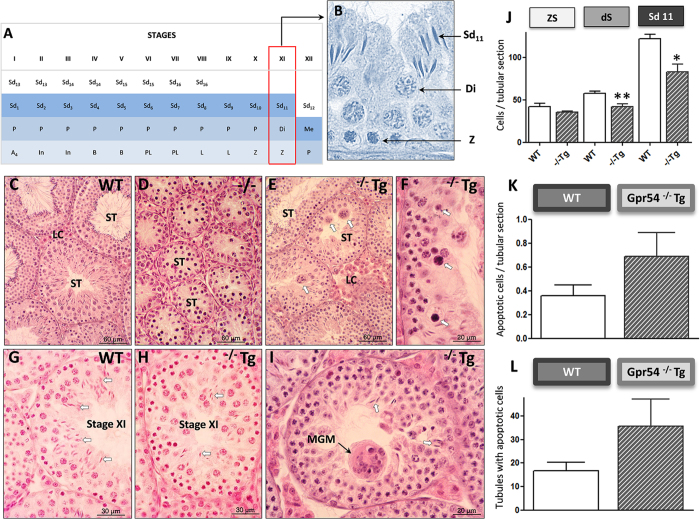
Alterations in testicular ultrastructure in the Gpr54^−/−^Tg rescued male mouse. A schematic drawing of the cycle of the seminiferous epithelium is presented in panel (**A**), while a representative image of germ cell types quantified in stage XI is shown in panel (**B**). In panels (**C**–**I**), representative testicular sections from WT, Gpr54^−/−^Tg rescued and Gpr54^−/−^ null female mice, stained with hematoxylin and eosin, are shown. WT male mice showed differentiated Leydig cells (*LC*) and seminiferous tubules (*ST*) with complete spermatogenesis (see **C**). In contrast, Gpr54^−/−^ null mice presented undifferentiated Leydig cells and small seminiferous tubules (ST), with arrested spermatogenesis (see **D**). In turn, Gpr54^−/−^Tg rescued mice showed differentiated Leydig cells (*LC*), and seminiferous tubules (*ST*) with complete spermatogenesis, but these displayed reduced diameter (see **E**), frequent multinucleate (*arrows* in **E**) and apoptotic (*arrows* in **F**) germ cells, as well as reduced numbers of elongated spermatids (*arrows*) in stage XI, when compared with wt mice (see **G**,**H**). In panel (**I**), a higher magnification is presented of multi-nucleate germ cells (*MGC*), corresponding to degenerating spermatids, and scarce elongated spermatids (*arrows*). In addition, quantitative analyses, assessing the number of primary spermatocytes at zygotene (*Z*) or diakinesis (*Di*), as well as stage-11 elongated spermatids (*Sd*_*11*_), per tubule cross-section at stage XI of the seminiferous cycle is presented in panel (**J**). In addition, apoptotic cells per tubule cross-section and the proportion of seminiferous tubules showing apoptotic cells are presented as histograms in panels (**K**,**L**).

Testicular histological analyses of the above genotypes confirmed the discernible, intermediate phenotype of Gpr54^−/−^Tg males. Thus, while young adult WT mice displayed well-differentiated Leydig cells and seminiferous tubules with complete spermatogenesis (Fig. 4C), Gpr54^−/−^ null mice showed lack of differentiated Leydig cells and under-developed seminiferous tubules, with arrested spermatogenesis at the level of primary spermatocytes (Fig. 4D); yet, round spermatids were occasionally found in global KO mice. In contrast, Gpr54^−/−^Tg rescued mice of the same age showed differentiated Leydig cells and complete spermatogenesis (Fig. 4E). However, in 5 out of 8 (~65%) rescued mice, spermatogenic alterations were observed, consisting of abundant apoptotic (Fig. 4F) and multinucleated (symplasts; Fig. 4E,I) germ cells, decreased numbers of elongated spermatids (Fig. 4G,H,I) and disorganization of the seminiferous epithelium (Fig. 4F,I). Quantitative data of these alterations are shown in Fig. 4J–L: Gpr54^−/−^Tg male mice showed a higher proportion of seminiferous tubules with apoptotic cells and a higher number of apoptotic cells per tubule cross-section, although differences were not statistically significant due to the large variability in transgenic mice. In addition, the number of spermatocytes in diakinesis and elongated spermatids in stage XI was significantly decreased in Gpr54^−/−^Tg mice, whereas the number of zygotene spermatocytes was not different.

### Feedback regulation of gonadotropins in Gpr54^−/−^Tg rescued mice-Responses to GNX

**Figure 5:**
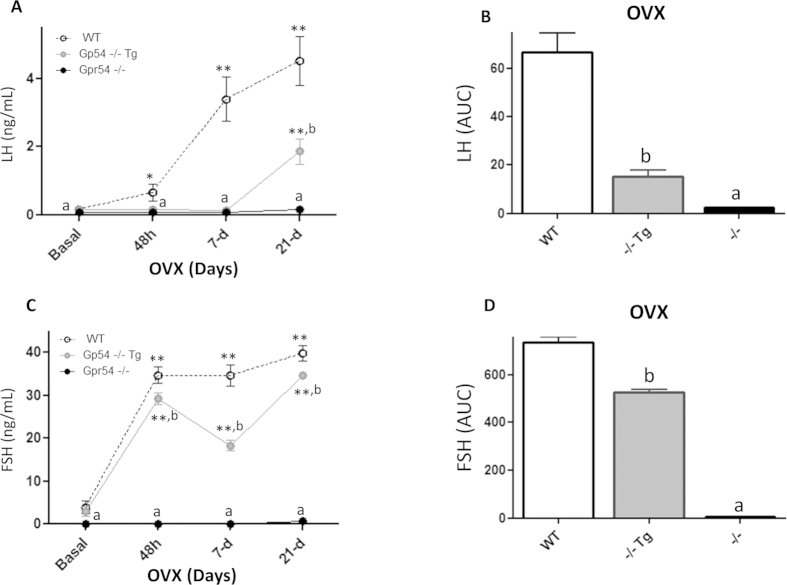
Perturbed negative feedback in the Gpr54^−/−^Tg rescued female mouse. Serum LH and FSH levels before and at different time-points after removal of gonadal feedback input by ovariectomy (OVX) were measured in adult WT, Gpr54^−/−^Tg rescued and Gpr54^−/−^ null female mice. In panel (**A**), serum LH levels at basal conditions and 48-hour, 7-day, 21-day after OVX are shown; integral levels over the study-period, calculated as AUC by the trapezoidal rule, as presented in panel (**B**). Similar analyses of changes in FSH levels following OVX in the three genotypes are presented in panels (**C**,**D**). **P < 0.01 vs. corresponding basal (pre-GNX) levels; **a**, **b** P < 0.05 vs. corresponding WT groups (ANOVA followed by Student-Newman-Keuls multiple range test).

To ascertain the roles of direct vs. indirect effects of kisspeptin on GnRH neurons in conveying the negative feedback effects of sex steroids, the time-course changes in LH and FSH levels were assayed in young adult Gpr54^−/−^Tg rescued mice at different time-points after GNX, and compared with those of WT and null mice, in both males and females. Gonadotropin levels were monitored before and 48-hour, 7-d and 21-d after removal of negative feedback signals to sex steroid withdrawal. Gonadotropin responses to GNX in female mice of the three genotypes are shown in Fig 5. In keeping with previous references, serum LH levels were elevated in WT females already 48-h after GNX and progressively increased thereafter, with maximal concentrations at 21-d after removal of gonadal secretions; in contrast, no increase in circulating LH was detected in Gpr54 null females, at any of the time points studied after GNX. Notably, LH responses to GNX were markedly attenuated in Gpr54^−/−^Tg rescued female mice, in which significant increases in serum LH levels were only detected at 21-d after GNX (Fig. 5A). Such a defective response manifested also in the integral LH secretory mass during the 21-d period following GNX, which was severely blunted (>75% reduction) in Gpr54^−/−^Tg rescued females (Fig. 5B). Roughly similar patterns were observed for FSH following GNX in the three genotypes, with partially attenuated FSH responses to GNX being detected in Gpr54^−/−^Tg rescued females, both in terms of time-course profiles (Fig. 5C) and integral secretory mass over the 21-d period following GNX (Fig. 5D). Yet, the degree of attenuation of FSH responses to GNX was much milder than for LH, with a significant ~25% reduction vs. WT levels in ovariectomized animals.

**Figure 6:**
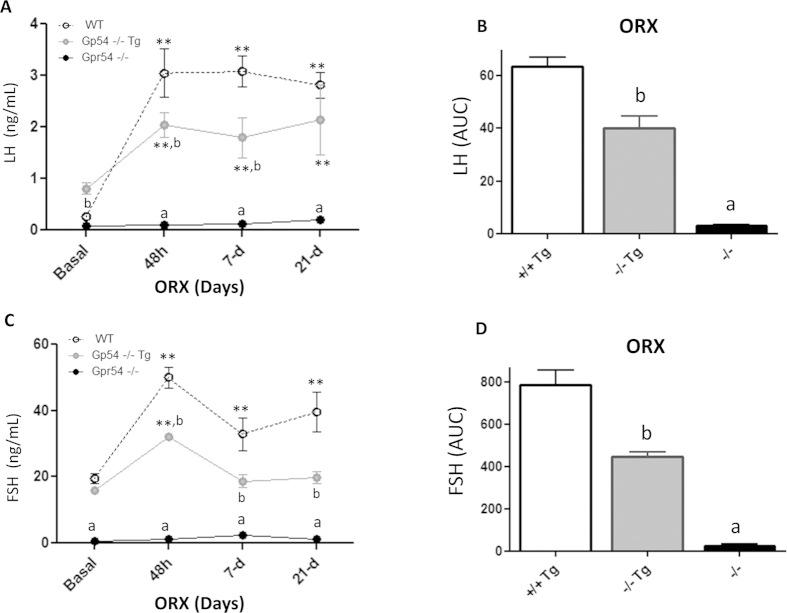
Perturbed negative feedback in the Gpr54^−/−^Tg rescued male mouse. Serum LH and FSH levels before and at different time-points after removal of gonadal feedback input by orchidectomy (ORX) were measured in adult WT, Gpr54^−/−^Tg rescued and Gpr54^−/−^ null male mice. In panel (**A**), serum LH levels at basal conditions and 48-hour, 7-day, 21-day after ORX are shown; integral levels over the study-period, calculated as AUC by the trapezoidal rule, as presented in panel (**B**). Similar analyses of changes in FSH levels following OVX in the three genotypes are presented in panels (**C**,**D**). **P < 0.01 vs. corresponding basal (pre-GNX) levels; **a**, **b** P < 0.05 vs. corresponding WT groups (ANOVA followed by Student-Newman-Keuls multiple range test).

Alike, gonadotropin responses to removal of gonadal feedback signals were also blunted in young adult Gpr54^−/−^Tg rescued male mice; yet, some differences in the patterns of gonadotropin responses to GNX were detected between sexes. Thus, in contrast to rescued females, LH levels were elevated at all time-points after orchidectomy, although the magnitude of such increase was lower than that observed in WT males, except for the latest time-point (21-d) studied (Fig. 6A). This resulted in a significant ~33% reduction in the integral LH responses to GNX over the 21-d period studied, as compared to WT males (Fig. 6B). Similarly, partially suppressed FSH responses to GNX were detected in Gpr54^−/−^Tg rescued males, although in this case the degree of attenuation was higher than in females of the same phenotype. In fact, GNX caused only a transient increase in serum FSH levels 48-h after GNX, which was not persistent at later time-points (Fig. 6C). In good agreement, integral FSH levels in adult Gpr54^−/−^Tg male mice over the 21-d period following GNX were only half of those detected in WT males (Fig. 6D). As in females, Gpr54^−/−^ null mice did not display elevations of LH or FSH levels at any time point after orchidectomy, in line with previous literature^11^.

### Hormonal responses to central activators of the gonadotropic axis in Gpr54^−/−^Tg rescued mice

To evaluate the relative importance of direct Gpr54 signaling in GnRH neurons for the stimulatory effects of different central regulators of the GnRH system, LH responses (as proxy marker of GnRH activation) to a number of agonists or antagonists of key central regulatory signals were evaluated in young adult (3–4 month-old) Gpr54^−/−^Tg rescued male mice, and compared with those of WT and null mice. Basal LH levels and responses to the different secretagogues were compared also between WT and Gpr54^+/+^Tg mice (i.e., WT mice harboring the Gpr54 transgene), as additional control. These analyses demonstrated that WT and Gpr54^+/+^Tg display similar basal LH levels (in both males and females), and roughly similar LH responses to different secretagogues, such as Kp-10 (that evoked LH responses that were even marginally greater in Gpr54^+/+^Tg than in WT mice) and different TK agonists (Suppl. Fig. 2). Altogether, these data validate the use of WT mice as control for our neuroendocrine tests and argue against the possibility that over-expression of the Tg *per se* might be a major contributor to the neuroendocrine phenotype of rescued mice. Of note, since basal LH levels were increased in Gpr54^−/−^Tg males, but significantly suppressed in Gpr54^−/−^ null mice, for analysis of data, not only absolute responses, but also relative responses to the different secretagogues, obtained after normalization by the corresponding basal LH concentrations, were compared among the different genotypes.

In a preliminary assay, young adult WT, Gpr54^−/−^Tg rescued and Gpr54 null mice were tested for LH responses to GnRH. As shown in Suppl. Fig. 3A, the three genotypes were responsive to GnRH, with similarly maximal absolute LH responses in WT and Gpr54^−/−^Tg males, thus demonstrating fully preserved pituitary responsiveness in rescued mice. Of note, blunted but significant LH responses to GnRH were detected in Gpr54 null mice, even after appropriate GnRH priming. Relative LH responses to GnRH (over corresponding basal levels) were not significantly different among the genotypes, although a trend for lower relative responses was detected in both Gpr54^−/−^Tg and Gpr54^−/−^ null mouse lines (Suppl. Fig. 4).

**Figure 7:**
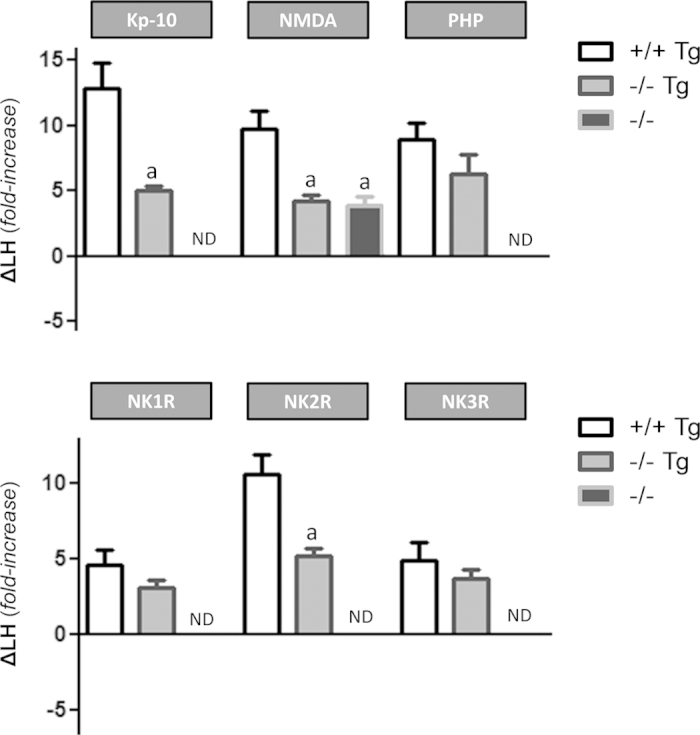
Perturbed relative responses to different central regulators in the Gpr54^−/−^Tg rescued mouse. Relative LH responses to different central regulators of the gonadotropic axis in adult WT, Gpr54^−/−^Tg rescued and Gpr54^−/−^ null male mice are shown. The animals were subjected to central (icv) injection of effective doses of kisspeptin-10 (Kp-10), NMDA (agonist of ionotropic glutamate receptors), PHP (antagonist of GABA-A receptors), or agonists of the tachykinin receptors, NK1R, NK2R and NK3R. Hormonal levels were assayed 15-min after icv administration of the compounds. Animals injected with vehicle (Veh) served as controls to set basal hormonal levels. Considering the notable differences in basal LH levels among the three genotypes, hormonal responses were normalized by the corresponding basal levels (set at 0 line), to express relative increments of LH secretion (ΔLH). **a** P < 0.01 vs. corresponding stimulated values in WT Gpr54+/+ mice (ANOVA followed by Student-Newman-Keuls multiple range test). ND: No increment detected.

In a first set of experiments, absolute and relative LH responses to Kp-10, the agonist of the subtype of glutamate ionotropic receptor, NMDA, and the antagonist of GABA-A receptors, PHP, were explored in adult WT, Gpr54^−/−^Tg and Gpr54^−/−^ null mice. Robust absolute LH responses to the three stimuli were detected in WT and rescued mice. In contrast, Gpr54 KO mice were irresponsive to Kp-10 (as expected) or the blockade of GABA-A receptors, while they retained partial responsiveness to NMDA (Suppl. Fig. 3B–D), in keeping with previous references^11^. In terms of relative responses, however, the stimulatory effects of Kp-10 (>12-fold increase) were severely attenuated in Gpr54^−/−^Tg rescued mice, while the relative increase in LH levels after blockade of GABA-A receptors was not different between WT and rescued males. In the case of NMDA stimulation, the robust 9-fold elevation of LH levels detected in WT was markedly (and equally) blunted in rescued and Gpr54 null mice (Fig. 7, *upper panel*).

In addition, absolute and relative LH responses to central administration of agonists to the three TK receptors, NK1R (GR73762), NK2R (GR64349), and NK3R (senktide), were evaluated in the three genotypes indicated above. Marked absolute LH responses were detected in adult WT and Gpr54^−/−^Tg rescued mice following central injection of the three TK agonists. In contrast, Gpr54 null mice failed to respond to any of the three TK stimuli (Suppl. Fig. 3E–G). Regarding relative responses, differences were detected between genotypes and the three TK receptor subtypes. Hence, the relative increases (~4–5 fold increase) in LH levels detected following central injection of NK1R and NK3R agonists were similar in WT and rescued mice. In contrast, NK2R activation in WT animals caused a robust 10-fold rise of serum LH levels, which was halved in Gpr54^−/−^Tg rescued mice (Fig. 7, *lower panel*).

## Discussion

The discovery of kisspeptins has substantially expanded our understanding of the mechanisms of neuroendocrine control of the HPG axis^1^,^20^, not only because these are key players in the regulation of puberty and fertility, but also because their identification has surfaced novel interactions with other important regulators of the reproductive system, acting at central and peripheral levels^21^. In this context, elucidation of the primary sites and mode of action of kisspeptins is not banal, but rather poses considerable translational interest, as it might help to refine current strategies for the pharmacological manipulation of the HPG axis in conditions ranging from precocious puberty to polycystic ovary syndrome and infertility.

Compelling evidence has documented that kisspeptins ultimately modulate the output of GnRH neurons to conduct their potent stimulatory effects on the reproductive system. Recent evidence from functional genomics supports the view, anticipated on the basis of initial expression and electro-physiological studies, that these actions are conducted directly at the level of GnRH neurons, since selective elimination of Gpr54 from GnRH cells recapitulates the hypogonadal phenotype of global Gpr54 null animals^19^,^22^, whereas rescue of Gpr54 signaling selectively in GnRH neurons appears to be sufficient to attain puberty and fertility^19^. While this contention is not refuted (but rather supported) by our current data, our detailed neuroendocrine analyses of a mouse line with GnRH neuron-specific Gpr54 rescue unveils a spectrum of reproductive deficits that argues against an unimodal mechanism of action of kisspeptins (namely, only direct effects in GnRH neurons) and is compatible with effects of kisspeptins at other central and peripheral elements of the HPG axis, in line with previous, as yet fragmentary and inconclusive evidence.

Mice of both sexes with restricted expression of Gpr54 selectively in GnRH-expressing cells did show clear alterations in different reproductive parameters, including basal gonadotropin (LH) levels, gonadal micro-architecture and gonadotropin responses to removal of negative feedback by gonadal secretions. Admittedly, the reproductive phenotype of our Gpr54^−/−^Tg rescued mice might theoretically derive, at least partially, from the aberrant expression of Gpr54 under the GnRH promoter, which may result in perturbed levels and/or regulation of Gpr54 expression in GnRH neurons, as well as residual mistargeted expression of Gpr54 outside hypothalamic GnRH neurons. A significant contribution of such phenomena, however, seems unlikely, not only in light of previous validation experiments that documented predominant, selective re-expression of Gpr54 in GnRH neurons in our Gpr54^−/−^Tg mouse line^19^, but also because of our thorough neuroendocrine characterization of Gpr54^+/+^mice harboring the Gpr54 transgene. These Gpr54^+/+^Tg mice displayed hormonal profiles, in terms of basal gonadotropin levels and major responses to GnRH regulators, which were grossly similar to those of WT mice. Admittedly, LH responses to Kp-10 were even marginally augmented in Gpr54^+/+^Tg vs. WT mice. Yet, this phenomenon is likely due to the summation of endogenous and Tg-driven over-expression of Gpr54 in GnRH neurons in this genotype (that does not occur in Gpr54^−/−^Tg animals, which are devoid of Gpr54 expression from the endogenous locus) and conclusively demonstrates the preserved functionality of Gpr54 signaling in our Tg model. All in all, these data support the view that over-expression of Gpr54 under the GnRH promoter is not *per se* a major causative factor for the reproductive alterations of our rescued knock-in line, and suggest the existence of central and peripheral defects due to the lack of Gpr54 signaling outside GnRH cells.

At the peripheral level, clear ovarian and testicular alterations were detected in Gpr54^−/−^Tg mice, in the absence of defective basal gonadotropin levels; if any, rescued males displayed elevated circulating LH concentrations in face of normal-to-low T secretion, which might be compatible with a primary testicular failure in males without kisspeptin signaling outside GnRH cells. In good agreement, a number of ultra-structural alterations were detected in the gonads of young adult Gpr54^−/−^Tg rescued mice, including lower numbers of spermatocytes in diakinesis and elongated spermatids, and trends for higher numbers of apoptotic cells in seminiferous tubules. Likewise, subtle but detectable alterations in ovarian histology were detected in Gpr54^−/−^Tg rescued females, which displayed features reminiscent of premature ovarian ageing. All in all, these findings extend and complement recent findings on the potential role of local kisspeptin signaling in the control of testicular and ovarian physiology. On the latter, we have recently provided evidence that kisspeptin signaling in the oocyte promotes survival and prevents premature ovarian failure^15^,^16^. Our present findings are compatible with accelerated senescence of the ovaries of mice without Gpr54 expression outside GnRH neurons, therefore suggesting the relevance of local ovarian kisspeptin actions. It must be stressed, however, that our data cannot rule out the contribution of perturbations in the dynamics of gonadotropin secretion due to central deficits in our Gpr54^−/−^Tg rescued model, as discussed below.

Indeed, besides gonadal alterations, mice with selective re-expression of Gpr54 exclusively in GnRH cells displayed perturbed neuroendocrine responses compatible with central defects in neuronal networks modulating GnRH neurosecretion. Thus, gonadotropin responses to withdrawal of the negative feedback actions of gonadal steroids were severely blunted in male and female Gpr54^−/−^Tg rescued mice, which exhibited an intermediate phenotype in between WT and global Gpr54 null animals. Thus, the post-OVX rise in circulating LH levels was dramatically suppressed in rescued females (only 25% of WT), whereas the elevation in FSH levels was blunted by one-forth. Similarly, the elevation of serum LH and FSH levels following ORX was blunted by 35–50% in Gpr54^−/−^Tg rescued males. Admittedly, gonadotropin responses to sex steroid replacement after GNX were not tested in our Gpr54^−/−^Tg mouse line. Yet, considering that sex steroids were not overtly suppressed in this rescued model, as evidenced by direct measurements in males or indirect estimations (e.g., uterus weight) in females, it is tenable that the defective responses to gonadal hormone withdrawal observed in Gpr54^−/−^Tg mice are due to the impairment of the central networks conveying the negative feedback actions of sex steroids ultimately to GnRH neurons. In this context, it has been conclusively documented that Kiss1 neurons in the arcuate nucleus play a key role in negative feedback regulation of gonadotropin secretion^11^,^23^. Our present data suggest that at least part of such regulatory action is conducted via Gpr54 signaling outside GnRH neurons, which might modulate other afferents to GnRH cells. While accurate estimation of the actual *weight* of such indirect actions might be difficult just on the basis of our serial LH and FSH measurements, the notable magnitude of the deficit in post-GNX responses seen in our Gpr54^−/−^Tg rescued model strongly suggests the contribution of kisspeptin signaling outside GnRH neurons in mediating this phenomenon.

The analysis of relative LH responses (as surrogate marker of GnRH secretion *in vivo*) to different regulators of the gonadotropic system also suggested changes in the functionality of central components of the reproductive axis in Gpr54^−/−^Tg rescue mice. Of note, such changes cannot be attributed merely to insufficient rescue of Gpr54 expression in GnRH neurons, as according to previous electrophysiological data, the capacity of kisspeptin to activate GnRH neurons was fully preserved in Gpr54^−/−^Tg mice^19^. In fact, contrary to global Gpr54 null mice, rescued mice retained robust responsiveness to the different secretagogues tested, stressing the importance of direct kisspeptin signaling in GnRH neurons for the manifestation of secretory responses to a wide variety of stimuli, ranging from glutamate to various TKs, as well as GABA signaling inhibition and kisspeptin itself. On the latter, despite robust absolute responses, relative LH responses to Kp-10 were attenuated in Gpr54^−/−^Tg rescued mice, suggesting that a component of the central stimulatory effects of kisspeptins on GnRH neurons *in vivo* might be conveyed via intermediary afferents, which, according to recent literature, could involve glutamate and NO transmission^8^,^10^, among others. It must be stressed, though, that differences in basal levels between phenotypes do have an impact in the magnitude of relative responses, calculated as fold-increase over those non-stimulated levels. Hence, part of the above attenuation, as well as of other changes in relative responses to the various secretagogues might be related with the elevation of basal LH levels in Gpr54^−/−^Tg male mice. In fact, relative LH responses to GnRH, which acts down-stream kisspeptin actions, tended to be decreased also. Nonetheless, we believe that relative responses are very informative and helpful for the neuroendocrine characterization of our model, as they allow the transversal comparison of responses to a given stimulus between genotypes, in spite of clear differences in basal LH levels, as well as the normalized comparison of responses to different stimuli within the same genotype.

Our *in vivo* data is the first to document that the potent stimulation following central inhibition of GABA-A receptors is totally eliminated in global Gpr54 KO animals, suggesting that kisspeptin signaling is mandatory for such effect. Recent evidence, based on electrophysiological recordings, has documented the existence of GABAergic transmission to Kiss1 neurons in the hypothalamus, whose magnitude and postsynaptic effects are modulated as function of the time of the day and estrogen background in female mice^24^. Our data provide conclusive evidence for the physiological relevance of such transmission. Furthermore, our analysis of the relative responses to the GABA-A antagonist in the three genotypes demonstrates that, while LH responses are blunted in global KO animals, the Gpr54^−/−^Tg rescued mice display fully preserved (absolute and relative) LH secretory responses to GABA receptor blockade, suggesting that this action is completely dependent on kisspeptin signaling in GnRH neurons. This is in clear contrast with responses to NMDA, which were only partially attenuated in global Gpr54 null mice, suggesting that a fraction of the stimulatory effects of ionotropic glutamate receptors is kisspeptin-independent. Of note, relative LH responses to NMDA were roughly similar between Gpr54 KO and Gpr54^−/−^Tg rescued mice, suggesting that, to a significant extent, kisspeptin-dependent actions of glutamate signaling would be conveyed indirectly to GnRH neurons, i.e., via kisspeptin receptors outside this cell type.

Compelling evidence accumulated in recent years has documented that the stimulatory effects of NKB, acting via NK3R, on GnRH neurons is dependent on the modulation of kisspeptin output onto GnRH neurons^11^,^21^. More recently, the ability of other TKs, such as the ligands of NK1R and NK2R, to activate Kiss1 neurons and to induce kisspeptin-dependent stimulation of GnRH/gonadotropin secretion has been documented^12^. Our present data significantly extend those previous observations. In one hand, global Gpr54 KO mice were totally irresponsive to the agonists of the TK receptors, NK1R, NK2R and NK3R, confirming that preserved kisspeptin signaling is mandatory for the stimulatory effects of TKs on GnRH neurons to occur. More interesting, our analyses of relative LH responses to the above TK agonists revealed notable differences among receptor subtypes. Thus, while activation of the NKB receptor, NK3R, and NK1R evoked similar responses in WT and Gpr54^−/−^Tg mice, relative LH secretory responses to the agonist of NK2R were significantly attenuated in Gpr54^−/−^Tg rescued mice (∼50% of WT controls). These data suggest that whereas the stimulatory effects of NKB and the ligands of NK1R, such as substance P, are completely dependent on kisspeptin signaling directly in GnRH neurons, the releasing effects of NK2R on GnRH might require the contribution of kisspeptin receptors located outside GnRH neurons. Thus, while preserved Gpr54 signaling is mandatory for the stimulatory effects of all TKs on GnRH neurons, our data strongly suggest differences in the sites of action whereby TKs impinge on kisspeptin pathways to modulate GnRH neurosecretion.

In sum, we provide herein data compatible with a physiological role of kisspeptin signaling at sites of the HPG axis other than GnRH neurons for the fine control of the reproductive axis. While our data do not refute, but rather confirm, that primary actions of kisspeptins directly on GnRH neurons are dominant for the reproductive role of this neuropeptide system, our current results surface, using a validated physiological setting, that such direct actions appear to be insufficient to allow complete preservation of proper functionality of gonadotropic axis, as evidenced by altered gonadotropin secretion, perturbed negative feedback regulation and diminished relative responses to some key central regulators. These results illuminate the complex mode of action of kisspeptin in the control of the HPG axis, whose knowledge may help to define improved approaches for the management of reproductive disorders.

## Methods

### Generation and genotyping of Gpr54^−/−^Tg rescued mice

A GnRH cell-specific Gpr54 expressing (rescued) mouse line was recently generated in a C57BL/6 background, using BAC transgenesis (see Suppl. Fig. 1A), and validated by the groups of G. Schuzt and M. Kirilov (German Cancer Research Center, Heidelberg, Germany) and A.E. Herbison (Centre of Neuroendocrinology, University of Otago, NZ), as extensively described elsewhere^19^. In this mouse line, selective (re)expression of Gpr54 in GnRH cells, over a Gpr54 null background, was achieved^19^. Of note, since the original Gpr54^−/−^ mouse used to generate the rescued mouse line is mainly a translational knockout, ultra-sensitive PCR evaluation of *Gpr54* transcript expression in different reproductive tissues was not considered for validation purposes^19^. Yet, detailed functional validation experiments had previously documented effective rescue of kisspeptin signaling in GnRH neurons, but complete ablation of Gpr54 expression/functionality in other reproductive tissues, such as the gonads and the pituitary^19^. This mouse line was transferred to our laboratory by Drs. Schuzt and Kirilov for extensive phenotypic and neurohormonal studies. Genotyping was conducted by PCR analyses on isolated genomic DNA from tail biopsies. The primers used for detection the wild-type (WT) allele were: T1003E: 5′-GCC TAA GTT TCT CTG GTG GAG GAT G‐3′ and T1003TE: 5′-CGC GTA CCT GCT GGA TGT AGT TGA C‐3′, while the primer pair used to detect the mutated allele was: NeoT: 5′-GGG TGG GAT TAG ATA AAT GCC TGC TCT-3′ and T1003TE: 5′-CGC GTA CCT GCT GGA TGT AGT TGA C‐3′. To evaluate the presence of the Gpr54 transgene, whose expression was directed to GnRH cells, the primers used were GnRH_F: 5′-GGT TTC AGG GAA CCC AAA TTA-3′ and Gpr54_R: 5′-ACC AAT GAG TTT CCG ACC AG-3′. PCR amplification was carried out using the following protocol: denaturing for 5 min at 95 °C followed by 30 cycles consisting of denaturing at 95 °C for 30 sec, annealing at 63 °C/57.5 °C for 30 sec and extension at 72 °C for 1 min, using an iCycler iQ-thermal cycler (Bio-Rad laboratories, Hercules, CA). Samples were subsequently analyzed on 2% agarose gels. A 253-bp fragment was generated by amplification of the wt allele whereas the mutant allele resulted in a 455-bp PCR fragment. A 439-bp fragment was generated by amplification of Gpr54 transgene (see Suppl. Fig. 1B). Thus, four genotypes were generated/considered for analysis: Gpr54^+/+^(WT), Gpr54^+/+^Tg, Gpr54^−/−^ (KO) and Gpr54^−/−^Tg (rescued). Given the similarities in profiles and responses between WT and Gpr54^+/+^Tg groups, WT mice are systematically presented as control genotype, except otherwise stated.

### Drugs

Mouse kisspeptin-10 (Kp-10) was obtained from Phoenix Pharmaceuticals Ltd (Belmont, CA). GnRH and the agonist of ionotropic glutamate receptors, N-methyl-D-Aspartate (NMDA, M3262), were purchased from Sigma Chemical Co. (St. Louis, MO). The agonists of the tachykinin receptors, NK1R (GR 73632), NK2R (GR 64349) and NK3R (senktide), as well as the antagonist of GABA-A receptor (PHP 501 trifluoroacetate) were purchased from Tocris Bioscience (Bristol, UK). Human choriogonadotropin (hCG; Profasi), as super-agonist of LH receptors, was obtained from Serono (Madrid, Spain). The drugs were dissolved in saline (0.9% NaCl), except for PHP 501, which was dissolved in 5% DMSO. Doses and timings for hormonal analyses were selected on the basis of previous studies^11^,^25^,^26^.

### Tissue processing and histological analyses

Histological analyses were applied to gonadal tissue from Gpr54^+/+^(WT), Gpr54^−/−^Tg (rescued) and Gpr54^−/−^ (KO) mice. Ovaries (including the oviduct and the tip of the uterine horn) and testes were fixed for at least 24-h in Bouin solution, and subjected thereafter to dehydration and embedded in paraffin wax. Serial (7 μm-thick) sections were cut, stained with hematoxylin and eosin, and evaluated under the microscope, using previously validated procedures in our group^27^,^28^.

## Experimental design

### General procedures

All the experimental protocols were approved by the Córdoba University Ethical Committee of animal experimentation and conducted in accordance with the European Union guidelines for use of experimental animals. For hormonal analyses, blood samples (200 μl) were obtained using standard procedures, routinely running in our laboratory^11^,^29^,^30^,^31^, by jugular venipuncture before (basal) and 15-min after injection of the compounds. For specific neuroendocrine tests, protocols of intracerebroventricular (icv) administration of selected compounds (Kp-10, GR 73632, GR 64349, senktide, PHP 501) were implemented, as described elsewhere^11^,^29^,^31^. In addition, peripheral (intraperitoneal; ip) injection of a single bolus of GnRH and blood sampling, at basal conditions and 30-min after peptide administration, was also conducted. Adult (3- to 4-mo-old) male mice of the Gpr54^+/+^, Gpr54^+/+^Tg, Gpr54^−/−^Tg (rescued) and Gpr54^−/−^ genotypes were used. To allow delivery of drugs into the lateral cerebral ventricle, the cannulae were lowered to a depth of 2 mm beneath the surface of the skull, with an insert point at 1 mm posterior and 1.2 mm lateral to bregma^11^. To exclude the possibility that defective gonadotropin responses to the various stimuli may stem from insufficient pituitary responsiveness to GnRH due to its low endogenous tone, which is characteristic of Gpr54-null animals, Gpr54^−/−^ mice were subjected to a protocol of GnRH priming during 2 d before each neuroendocrine test, to heighten pituitary responsiveness, in keeping with our previous reference^11^. The priming protocol, which was adapted from previous literature in other species^32^, consisted of five successive ip boluses of a low dose of GnRH (0.15 μg/each), with the following schedule: at 10:00 h, 17:00 h, and 23:50 h on d 1; at 08:00 h and 16:00 h on d 2. The other genotypes, including Gpr54^−/−^Tg (rescued) mice, were submitted to a similar protocol of five ip injections (but of physiological saline). Neuroendocrine tests were conducted between 09:00 h and 11:00 h of the following day.

### Study 1

#### Reproductive maturation and function of the Gpr54^−/−^Tg (rescued) female mouse-Major features

Major reproductive phenotypic (presence of vaginal opening, ovarian weights) and hormonal (LH and FSH) parameters were measured in female mice. In addition, estrous cyclicity was monitored by daily vaginal cytology, for a period of 30 days (corresponding to ~7 complete estrous cycles), and ovarian ultra-structure was analyzed in adult (3–4 month old) mice of the three genotypes: WT, rescued and KO (n = 5; except for KO = 3). In the ovaries of WT and Gpr54^−/−^Tg mice, the number of corpora lutea of the current cycle per ovary (that corresponds to the number of oocytes released during the last ovulation) was determined by scoring all serial sections. Additionally, ovarian ultra-structure was analyzed in WT and Gpr54^−/−^Tg mice (n = 5) at incipient ageing (12-mo-old animals).

### Study 2

#### Reproductive maturation and function of the Gpr54^−/−^Tg (rescued) male mouse-Major features

Major reproductive phenotypic (presence of preputial separation, testis and epididymis weights) and hormonal (LH, FSH and testosterone) parameters were measured in male mice. In addition, *ex vivo* testosterone secretion, both at basal conditions and after stimulation with an effective dose of hCG, was measured using static incubations testicular explants from Gpr54^+/+^, Gpr54^−/−^Tg (rescued) and Gpr54^−/−^ mice. Gpr54^−/−^ mice were subjected to a protocol of hCG (Lepori-Farma) priming *in vivo* before incubation; this priming protocol consisted of one daily ip bolus of hCG (5UI/mouse/day) for 5 days. WT and Gpr54^−/−^Tg mice were submitted to a similar protocol, but with injections of physiological saline. Procedures for incubation of testicular samples were as described in detail elsewhere^25^,^27^. Briefly, after decapitation of the animals, testes (n ≥ 10 per group) were removed immediately, de-capsulated and placed in scintillation vials in a Dubnoff shaker at 32 C with constant shaking (60 cycles/min), under an atmosphere of 95% O2/5% CO2. After 60 min of pre-incubation with DMEM (Lonza), the media were successively replaced by fresh medium or medium containing hCG (10 IU/ml), and tissue incubations were further conducted for 90-min, when samples from the media were collected for hormone determinations. The levels of testosterone in the media are expressed as normalized values per gram of incubated tissue. Finally, testicular histology was analyzed in adult (3–4 month old) mice of the three genotypes: WT, rescued and KO (n = 5 WT, 8 rescued & 3 KO). In morphometric analyses, the proportion of seminiferous tubules showing apoptotic cells and the number of apoptotic cells per tubule cross-section were determined in 50 tubular sections per animal. The number of primary spermatocytes in zygotene and diakinesis and the number of elongated spermatids in phase 11 of the spermiogenesis in stage XI of the cycle were determined in 25 tubular cross-sections per animal. Stage XI was selected because it is easily recognizable in hematoxylin-eosin stained sections, and germ cell types can be easily counted. Changes in the number of these cell types reflect cumulative cell death during previous stages of spermatogenic cycle.

### Study 3

#### Feedback regulation of gonadotropins in Gpr54^−/−^Tg (rescued) mice-Responses to GNX

The time-course of changes in the circulating levels of both gonadotropins, LH and FSH, in response to gonadectomy (GNX) was explored in male and female mice, of the three genotypes: Gpr54^+/+^, Gpr54^−/−^Tg (rescued) and Gpr54^−/−^. Groups of adult male and female mice (n ≥ 6) were subjected to bilateral orchidectomy (ORX) or ovariectomy (OVX) via abdominal route, in keeping with previous references^11^. Blood samples were obtained before, and at 48-h, 7-d and 21-d after GNX.

### Study 4

#### Hormonal responses to central activators of the gonadotropic axis in Gpr54^−/−^Tg (rescued) mice

Hormonal (LH) responses to known stimulators of GnRH and/or gonadotropin secretion were studied in Gpr54^+/+^(WT), Gpr54^−/−^Tg (rescued) and Gpr54^−/−^ (KO) male mice. Basal LH levels and responses to the different secretagogues were compared between WT and Gpr54^+/+^Tg mice (i.e., WT mice harboring the Gpr54 transgene), as additional control. For neuroendocrine test, the mice were injected with effective doses of GnRH, kisspeptin-10 (Kp-10), NMDA or the antagonist of GABA-A receptor (PHP-501), and blood samples were obtained at 15-min after administration of the compounds (in case of GnRH, 30-min after injection, due to its ip route of injection). In addition, similar neuroendocrine tests were conducted to assess LH responses to central injection of effective doses of agonists of the three tachykinin receptors, GR 73632 (NK1R), GR 64349 (NK2R), and senktide (NK3R). The doses, routes of administration and group sizes for these neurohormonal tests were as follows: GnRH, 0.25 μg/mouse in 100 μl/ip, n = 8 (except for Gpr54^−/−^Tg, n = 4); Kp-10, 50 pmol/mouse in 5 μl/icv, n = 13; NMDA, 1 nmol/mouse in 5 μl/icv, n = 7; PHP, 0.5 nmol/mouse in 5 μl/icv, n = 7; NK1R, 600 pmol/mouse in 5 μl/icv, n = 8; NK2R, 600 pmol/mouse in 5 μl/icv, n = 7; Senktide, 600 pmol/mouse in 5 μl/icv n = 7. Doses and routes of administration were selected in the basis of previous references^9^,^11^,^31^,^33^.

### Hormone measurements

Testosterone levels in static incubation media were measured using a commercial kit from MP Biomedicals (Costa Mesa, CA). All medium samples were measured in the same assay. The sensitivity of the assay was 1 ng/ml, and the intra-assay coefficient of variation was 4.5%. Serum gonadotropin levels were measured using a double-antibody method and RIA kits supplied by the National Institutes of Health (Dr. A.F. Parlow, National Hormone and Peptide Program, Torrance, CA). Rat LHI- 10 and FSH-I-9 were labeled with 125I using Iodo-gen tubes, as per manufacturer instructions (Pierce Chemical Co., Rockford, IL), in keeping with our previous references^11^. Hormone concentrations were expressed using reference preparations LH-RP-3 and FSH-RP-2 as standards. Intra-assay and inter-assay coefficients of variation (CV) were <8% and 10%, respectively.

### Presentation of data and statistics

All data are expressed as the mean ± SEM for each group. Unpaired Student *t*-tests or ANOVA followed by *post hoc* Student-Newman-Keuls tests were used to assess variation between experimental groups. The significance level was set at *P* ≤ 0.05. All analyses were performed with GraphPad Prism Software, Inc. (San Diego, CA).

## Additional Information

**How to cite this article**: León, S. *et al.* Direct Actions of Kisspeptins on GnRH Neurons Permit Attainment of Fertility but are Insufficient to Fully Preserve Gonadotropic Axis Activity. *Sci. Rep.* **6**, 19206; doi: 10.1038/srep19206 (2016).

## Supplementary Material

Supplemental Figures
