# Supplementary material for: Direct Actions of Kisspeptins on GnRH Neurons Permit Attainment of Fertility but are Insufficient to Fully Preserve Gonadotropic Axis Activity
Source: Sci Rep. 2016 Jan 12;6:19206. doi: 10.1038/srep19206 (PMC4709743; doi:10.1038/srep19206)
Supplement: Supplemental Figures [file srep19206-s1.pdf]

SREP-15-20365-A *(Revised-v2)*

SUPPLEMENTAL MATERIAL

Direct Actions of Kisspeptins on GnRH Neurons Permit Attainment of Fertility but are Insufficient to Fully Preserve Gonadotropic Axis Activity

Silvia León, Alexia Barroso, María J. Vázquez, David García-Galiano, María Manfredi-Lozano, Francisco Ruiz-Pino, Violeta Heras, Antonio Romero-Ruiz, Juan Roa, Günther Schutz[¶], Milen Kirilov[¶], Francisco Gaytan, Leonor Pinilla, Manuel Tena-Sempere

Department of Cell Biology, Physiology and Immunology, University of Córdoba; CIBER Fisiopatología de la Obesidad y Nutrición, Instituto de Salud Carlos III; Instituto Maimónides de Investigación Biomédica de Córdoba/Hospital Universitario Reina Sofia (IMIBIC/HURS), 14004 Córdoba, Spain; and [¶]Molecular Biology of the Cell I, German Cancer Research Center, Heidelberg, Germany

Legend to Supplemental Figures

Supplemental Figure 1: *Strategy for generation and genotyping of the Gpr54$^{-/-}$Tg rescued mouse*. In panel (**A**), a diagram depicting the strategy for BAC transgenesis, used to re-express Gpr54 (transgene: Tg) upon a Gpr54 null background is depicted (taken from Kirilov et al. 2013[19], with modifications). In addition, in panel (**B**), the strategy for genotyping of Gpr54$^{-/-}$Tg rescued mice, using a combination of primers to detect WT and mutant alleles of Gpr54, as well as the Gpr54 transgene is shown, together with representative images of PCR analyses of the different genotypes.

Supplemental Figure 2: *Hormonal profiles of WT Gpr54$^{+/+}$ and Gpr54$^{+/+}$Tg mice*. Basal LH levels in both control genotypes are shown, for male and female mice. In addition, absolute LH responses to Kp-10 and agonists of the different tachykinin receptors, NK1R, NK2R and NK3R, are presented. Hormonal levels were assayed 15-min after icv administration of the compounds. Animals injected with vehicle (Veh) served as controls. ** P<0.01 vs. corresponding vehicle-injected groups; **a** P<0.05 vs. corresponding WT groups (ANOVA followed by Student-Newman-Keuls multiple range test).

Supplemental Figure 3: *Absolute responses to different central regulators in the Gpr54$^{-/-}$Tg rescued mouse.* Absolute LH responses to different central regulators of the gonadotropic axis in adult WT, Gpr54$^{-/-}$Tg rescued and Gpr54$^{-/-}$ null male mice are shown. The animals were subjected to central (icv) injection of effective doses of kisspeptin-10 (Kp-10), NMDA (agonist of ionotropic glutamate receptors), PHP (antagonist of GABA-A receptors), or agonists of the tachykinin receptors, NK1R, NK2R and NK3R. Hormonal levels were assayed 15-min after icv administration of the compounds. Animals injected with vehicle (Veh) served as controls. ** P<0.01 vs. corresponding vehicle-injected groups; **a** P<0.05 vs. basal (vehicle) levels in WT Gpr54+/+ mice; **b** P<0.05 vs. stimulated levels in WT Gpr54+/+ mice (ANOVA followed by Student-Newman-Keuls multiple range test).

Supplemental Figure 4: *Relative responses to GnRH in the global Gpr54$^{-/-}$ null and the Gpr54$^{-/-}$Tg rescued mouse lines*. Relative LH responses to intra-peritoneal (ip) administration of an effective (0.25 µg) dose of GnRH in adult WT, Gpr54$^{-/-}$Tg rescued and Gpr54$^{-/-}$ null male mice are shown. Hormonal levels were assayed 30-min after ip injection of the peptide. Animals injected with vehicle served as controls to set basal hormonal levels. Hormonal responses were normalized by the corresponding basal levels (set at 0 line), in order to express relative increments of LH secretion (ΔLH). Data were analyzed by NOVA followed by Student-Newman-Keuls multiple range test. *P* values are shown for the Gpr54$^{-/-}$Tg rescued and Gpr54$^{-/-}$ null groups.

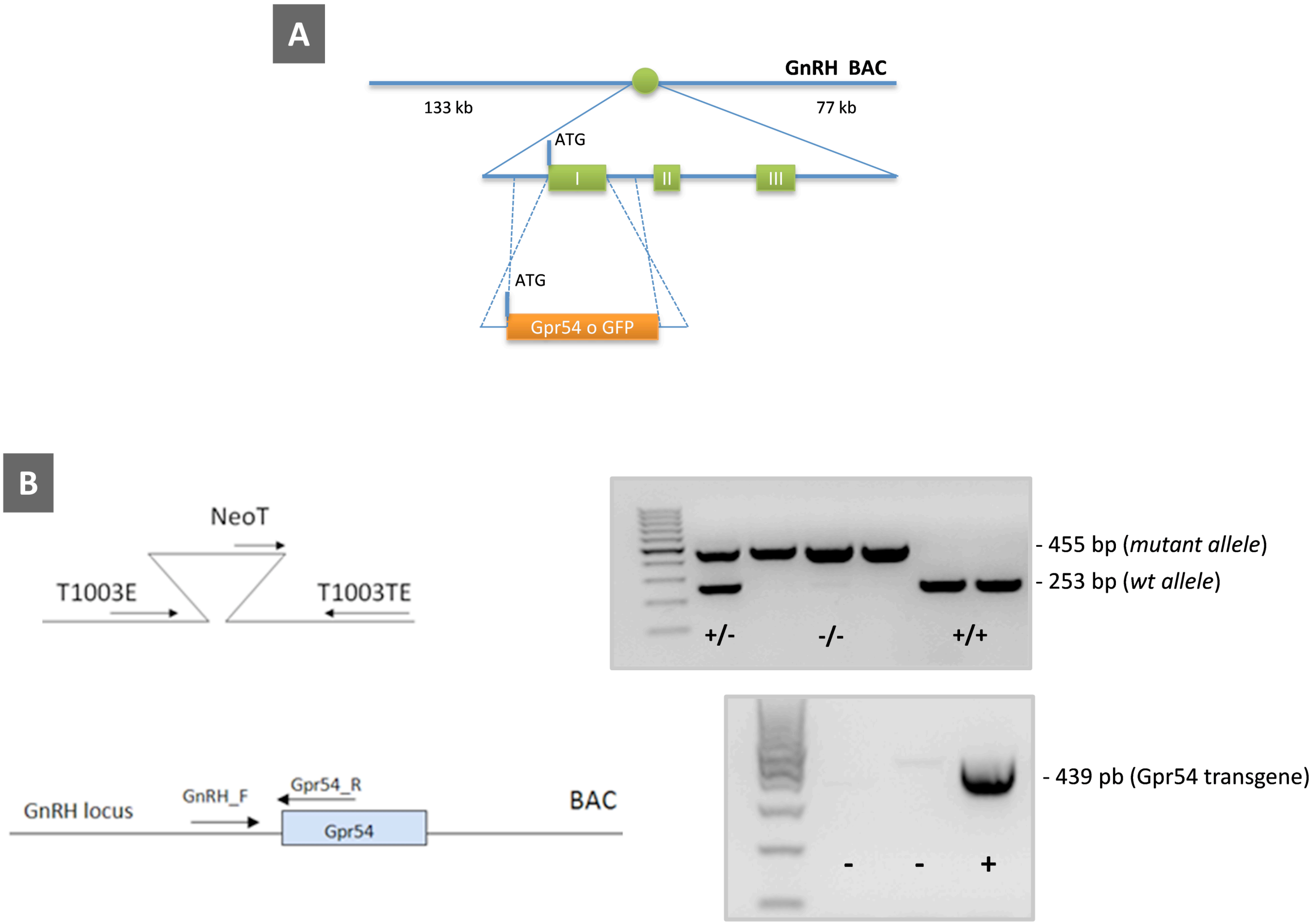
A
GnRH BAC
133 kb
77 kb
ATG
I
II
III
ATG
Gpr54 o GFP
B
NeoT
T1003E
T1003TE
+/-
-/-
+/+
- 455 bp (mutant allele)
- 253 bp (wt allele)
GnRH locus
GnRH_F
Gpr54_R
Gpr54
BAC
-
-
+
- 439 pb (Gpr54 transgene)

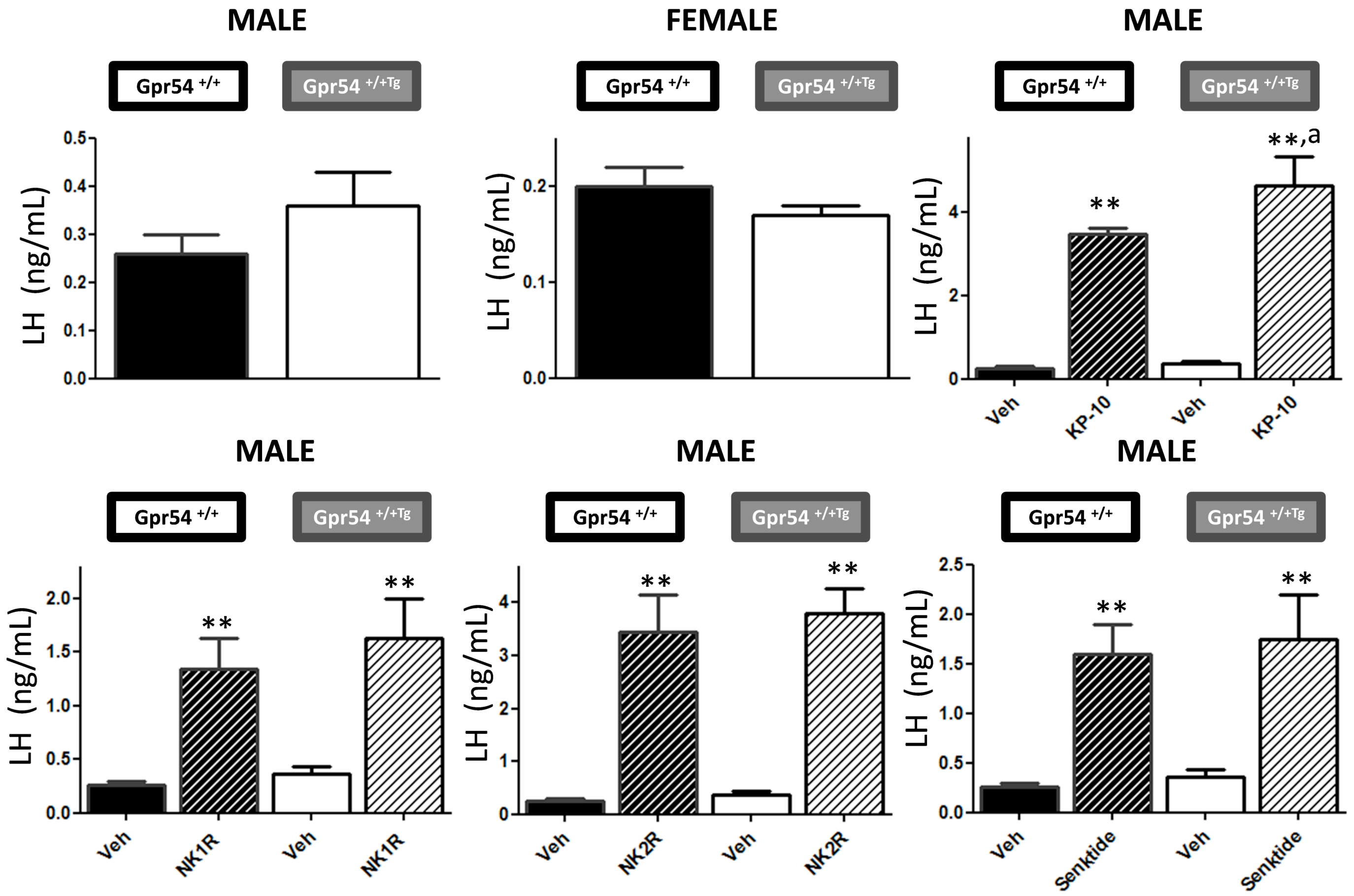

MALE
Gpr54 +/+
Gpr54 +/+Tg
LH (ng/mL)
FEMALE
MALE
Veh
KP-10
**
**,a
NK1R
NK2R
Senktide

MALE MICE

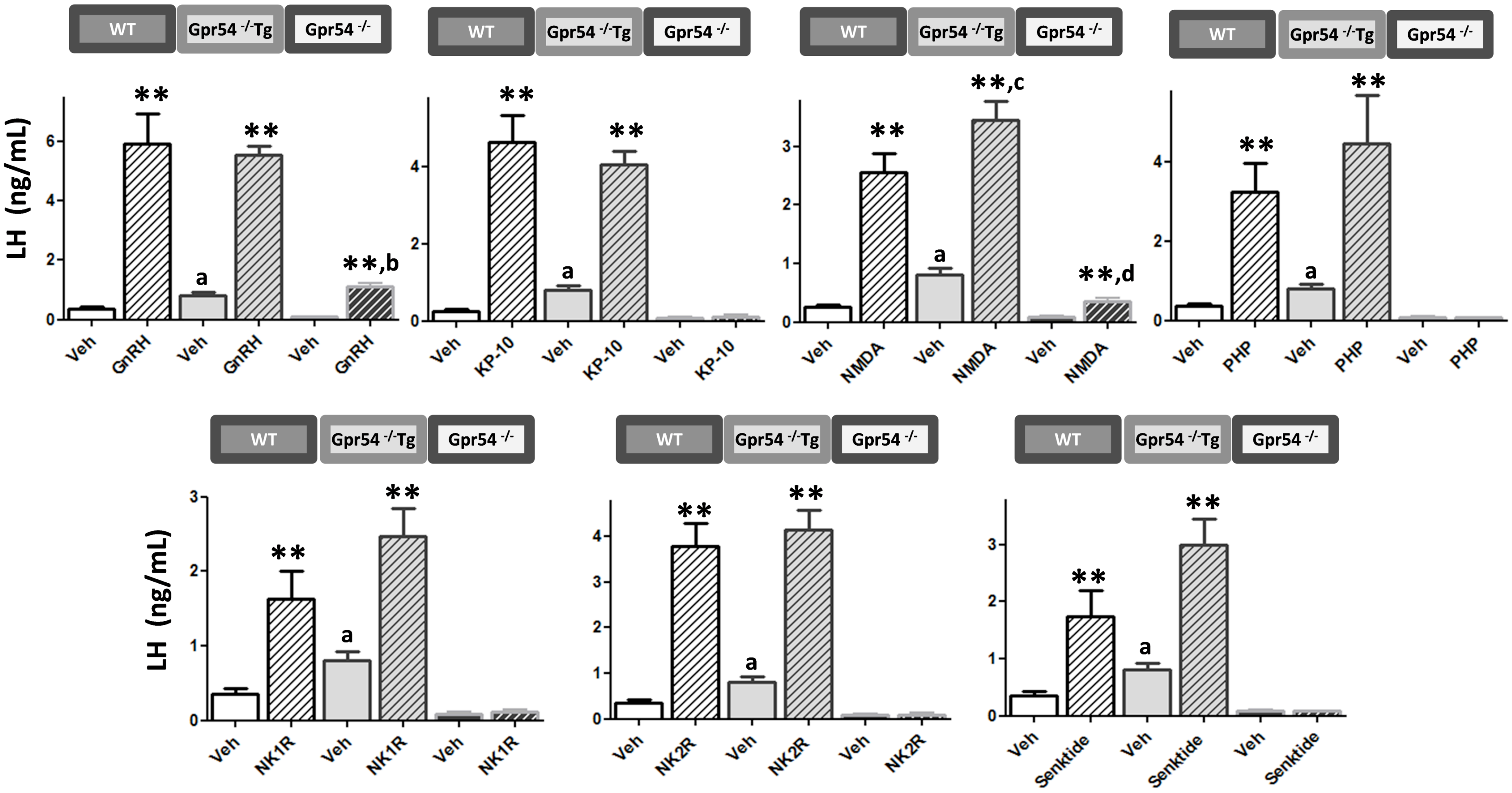

WT
Gpr54 -/-Tg
Gpr54 -/-
LH (ng/mL)
Veh
GnRH
KP-10
NMDA
PHP
NK1R
NK2R
Senktide
**
a
**,b
**,c
**,d

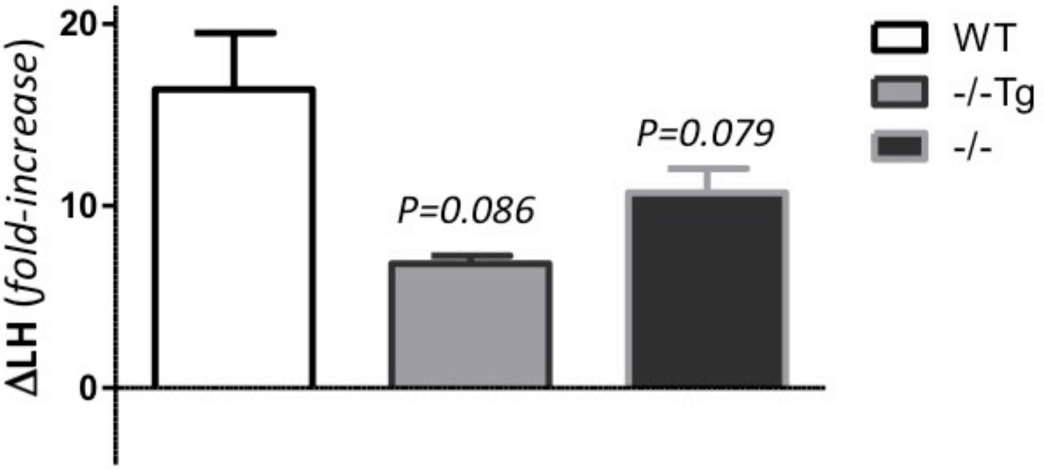

20
10
0
ΔLH (fold-increase)
P=0.086
P=0.079
WT
-/-Tg
-/-
